# Real-Time Monitoring of the In Situ Microfluidic Synthesis of Ag Nanoparticles on Solid Substrate for Reliable SERS Detection

**DOI:** 10.3390/bios11120520

**Published:** 2021-12-16

**Authors:** Niccolò Paccotti, Alessandro Chiadò, Chiara Novara, Paola Rivolo, Daniel Montesi, Francesco Geobaldo, Fabrizio Giorgis

**Affiliations:** 1 Department of Applied Science and Technology, Politecnico di Torino, C.so Duca degli Abruzzi 24, 10129 Torino, Italy; niccolo.paccotti@polito.it (N.P.); alessandro.chiado@polito.it (A.C.); paola.rivolo@polito.it (P.R.); daniel.montesi@polito.it (D.M.); francesco.geobaldo@polito.it (F.G.); fabrizio.giorgis@polito.it (F.G.); 2 Center for Sustainable Future Technologies @Polito, Istituto Italiano di Tecnologia, Corso Trento 21, 10129 Torino, Italy

**Keywords:** microfluidic synthesis, in situ monitoring, Ag nanostructures, porous silicon, SERS

## Abstract

A sharpened control over the parameters affecting the synthesis of plasmonic nanostructures is often crucial for their application in biosensing, which, if based on surface-enhanced Raman spectroscopy (SERS), requires well-defined optical properties of the substrate. In this work, a method for the microfluidic synthesis of Ag nanoparticles (NPs) on porous silicon (pSi) was developed, focusing on achieving a fine control over the morphological characteristics and spatial distribution of the produced nanostructures to be used as SERS substrates. To this end, a pSi membrane was integrated in a microfluidic chamber in which the silver precursor solution was injected, allowing for the real-time monitoring of the reaction by UV–Vis spectroscopy. The synthesis parameters, such as the concentration of the silver precursor, the temperature, and the flow rate, were varied in order to study their effects on the final silver NPs’ morphology. Variations in the flow rate affected the size distribution of the NPs, whereas both the temperature and the concentration of the silver precursor strongly influenced the rate of the reaction and the particle size. Consistently with the described trends, SERS tests using 4-MBA as a probe showed how the flow rate variation affected the SERS enhancement uniformity, and how the production of larger NPs, as a result of an increase in temperature or of the concentration of the Ag precursor, led to an increased SERS efficiency.

## 1. Introduction

A fine control over the parameters affecting the synthesis of metallic nanoparticles (NPs) is crucial for their application in biosensing [1,2,3]. Indeed, it is fundamental not only for techniques such as surface plasmon resonance (SPR) or surface-enhanced Raman scattering (SERS) [4,5], but also for those colorimetric detection mechanisms that depend on the NPs’ aggregation [6,7]. Actually, all these platforms require the coupling of plasmonic resonances to get an optical response that allows for performing the sensing of the species of interest. Then, a great effort has to be devoted to the optimization of features such as the size distribution and/or the shape of the nanostructures [8,9].

SERS spectroscopy has imposed itself as a reliable, label-free, and highly-sensitive technique, offering considerable advantages in several fields of application, particularly concerning the biological and biomedical sectors [10,11,12,13]. Moreover, the development of solid SERS platforms allowed for the overcoming of most of the stability issues characterizing the colloidal suspensions [14,15,16], even though some drawbacks exist concerning the difficulty and cost of fabrication [17,18,19]. It is, indeed, well-known that the optical properties of the SERS substrates are strongly related to the spatial distribution of the metal NPs, and in particular, to the size and distribution of the inter-particle gaps that generate the so-called hot-spots [20]. Unfortunately, the poor reproducibility of the synthesis processes of colloidal suspensions negatively affects the batch outcomes [21]. To overcome such a lack in the repeatability of the synthetic protocol, a microfluidic approach has been recently taken into account, aiming to achieve a better control over various synthesis parameters singled out to obtain the desired substrates. Both the heat and the mass transfer were boosted, thanks to the larger surface-to-volume ratio compared with a macroscopic synthesis environment [21,22,23,24]. In addition, the use of a microfluidic approach allowed for the study of the kinetics of the NPs’ growth mechanism in real time by means of optical spectroscopy [25].

Concerning the fabrication of nanostructured substrates with finely tuned morphological properties, single-phase microfluidic reactors have been used to achieve a sharp control over numerous parameters influencing the NPs’ synthesis, such as the concentrations of the reactants, temperature, and flow rate [14,26]. The combined variation of such parameters can be used to tune the size and the shape of the particles, therefore obtaining the desired plasmonic properties [25,27,28]. Moreover, the development of strategies for the in-flow in situ synthesis of NPs allows for the direct integration of such systems in sensing devices, and for on-site and real-time measurements [21,29,30,31,32,33,34].

Among the processes used for the fabrication of effective SERS substrates, the synthesis of noble metal nanostructures on porous silicon (pSi) membranes is an interesting option, thanks to the wide range of achievable morphologies, the high SERS efficiency in a wide range of excitation wavelengths [35,36], and the improved stability of the produced plasmonic nanostructures [37]. While the spontaneous deposition of metal NPs on pSi from metal salt solutions has been previously studied both on silicon [38,39,40] and flexible substrates [41,42], the microfluidic growth of such structures has not been extensively investigated.

In this work, a microfluidic approach was applied to the synthesis of Ag-NPs on porous silicon membranes supported on polydimethylsiloxane (PDMS), taking advantage of the reduction of the silver nitrate precursor by the active pSi surface. To this end, the pSi–PDMS membranes were integrated into a microfluidic chip, and UV–Vis transmittance spectroscopy was exploited to monitor, in real time, the evolution of the localized surface plasmon resonances (LSPRs) of the Ag-NPs during their growth, thus allowing for a fine-tuning of the synthesis reaction. At first, the effect of each synthesis parameter, such as the silver precursor concentration, temperature, and flow rate, on the morphological and optical properties of the Ag-pSi-based substrates was investigated. The morphology of the obtained samples was then carefully characterized by means of field emission scanning electron microscopy (FESEM) combined with a MATLAB routine for the image analysis of the collected micrographs. Lastly, the SERS efficiency and reproducibility of each sample was investigated exploiting 4-mercaptobenzoic acid (4-MBA) as a Raman probe in order to seek possible correlations between SERS performances, specific morphological characteristics, and spatial distributions of the silver NPs.

## 2. Materials and Methods

### 2.1. Porous Silicon Electrochemical Etching

The porous silicon (pSi) was obtained through the electrochemical etching of boron-doped silicon wafers (34–40 mOhm-cm resistivity, roughness <1 nm on the polished surface, Siltronix Silicon Technologies) in a 20:20:60 hydrofluoric acid (HF)/water/ethanol % v/v solution [43]. The anodization took place in a PTFE cell, applying a current density of 102 mA/cm^2^ for 7 s to produce a single porous layer with 68% porosity and 300 nm of thickness. These parameters were checked by simulation of the UV–Vis–NIR specular reflectance spectra of the membrane using the SCOUT 2.3 software (WTheiss Hardware and Software, Aachen, Germany). In order to detach the pSi monolayer from the silicon substrate, a second electrochemical attack was performed with a current density of 3.4 mA/cm^2^ for 35 s in a 4:4:92 HF/water/ethanol % v/v solution. After rinsing with ethanol, the pSi layer was transferred onto a 1 mM mM thick PDMS slice, obtained by the polymerization of a mixture of the PDMS oligomer and curing agent with a 10:1 ratio, cross-linked at 60 °C for 30 min [43]. The whole procedure took place in a temperature-controlled environment at 20 °C.

### 2.2. Microfluidic Synthesis of the Ag-NPs

**Scheme 1:**
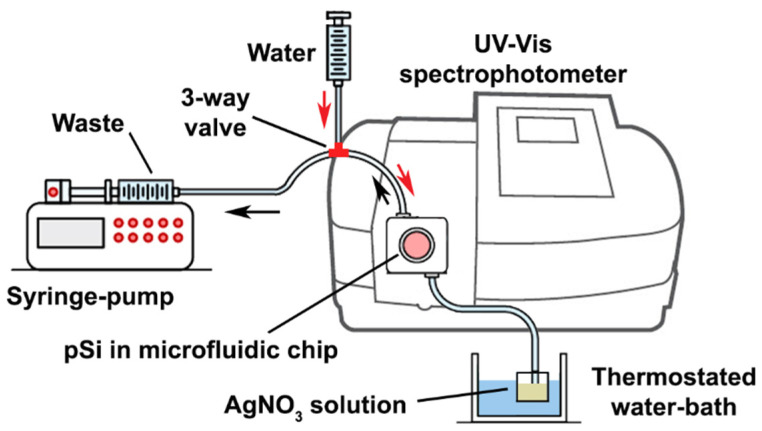
Scheme of the experimental set-up.

In order to investigate the effect of the synthesis parameters’ variation on the final size and distribution of the AgNPs, an in-flow synthesis protocol (hereby referred as “dynamic”) was designed. A scheme of the experimental setup used for the in situ analysis of the NPs’ growth is shown in Scheme 1 (a picture of the setup is shown in Figure S1 in Supplementary Materials). The 300-nm thick pSi membrane (diameter: 10 mm) was integrated into a microfluidic chip, according to the fabrication protocol previously developed by Novara et al. [42]. A single larger chamber was used, instead of a 4-chamber design, in order to match the dimensions of the light-source window of the spectrophotometer used for monitoring (see below). The cover was 4 mm high, with a thinner optical window of 3 mm. The active pSi surface, exposed in the microfluidic chamber, corresponds to an area of 78.54 mm^2^. Afterwards, the microfluidic chip was clamped to a homemade PMMA holder with an optical window centred on the pSi membrane (Figure S1b,c), and a syringe pump (11 plus, Harvard Apparatus, Holliston, Massachusetts) was used to drive the injection of a AgNO_3_ solution into the microfluidic chamber (back flow, black arrows in Scheme 1). The NPs’ growth was monitored in situ via UV–Vis transmittance spectroscopy using a Cary 5000 UV–Vis–NIR spectrophotometer (Agilent, Santa Clara, CA, USA). A scan between 300 nm and 700 nm was performed every minute, monitoring the evolution of the plasmonic dip. The flow rate was varied in a range between 0 and 3 mL/min, whereas the temperature of the precursor solution (20–60 °C) was controlled using a Julabo Corio CD thermostatic bath (Julabo, Seelbach, Germany). In detail, the reservoir containing the silver nitrate solution was immersed in the thermostatic bath. In order to avoid heat losses during the transfer of the solution, all the connections to the microfluidic chamber were thoroughly insulated. Moreover, an initial calibration test was performed by measuring the temperature inside the microfluidic chamber as water flowed in at different temperatures by means of a K-type thermocouple embedded inside the PDMS chip. This allowed us to compensate for any heat loss. The nanoparticle growth was finally quenched by injecting pure water into the microfluidic chamber after the saturation regime was reached (red arrows in Scheme 1). Each in-flow synthesis was carried out in a different microfluidic chamber, which was disassembled before the SERS measurements. All combinations of the synthesis conditions have been tested at least three times, and all the corresponding samples have been characterized as reported in the following. For the sake of comparison, the static condition (without continuously flowing the silver precursor solution) was also tested (hereby referred as “static”). To this end, an open pSi–PDMS membrane was attached to the wall of a plastic cuvette, which was filled with a silver nitrate solution whose concentration and temperature were also varied.

### 2.3. Field Emission Scanning Electron Microscopy (FESEM)

The FESEM morphological characterization was performed using a Zeiss SUPRA 40 microscope (Zeiss SMT, Oberkochen, Germany). The pSi/PDMS membranes, decorated with Ag-NPs, were recovered from the elastomeric microfluidic chip, and covered with a copper grid connected to the FESEM stub to mitigate the electron surface charge-up effect due to the poorly conducting thick PDMS layer. The typical imaging parameters were an acceleration tension between 2.5–5 kV, a working distance of 3.5 mm, and an aperture size of 20 μm.

### 2.4. Image Analysis

The morphology of a SERS substrate, especially concerning the size of the NPs and their inter-particle gap, plays a key role in determining the SERS efficiency. In order to characterize such features, a MATLAB routine devoted to the analysis of FESEM images, developed by Novara et al. [44], was employed. The script was then modified to remove the background noise, to adjust the image contrast, to separate the NPs’ aggregates, and to correct artefacts, thus allowing us to distinguish between dense and spread particles. In order to obtain the size distribution of the NPs, their shape was then approximated to a circular disk of comparable area. Inter-particle gaps were instead calculated exploiting the distance transform MATLAB operator, which identifies the distance of each background pixel from the closest object. Local maxima of the inter-object distances correspond to the semi-distance between two nanoparticles, and can, thus, be used to estimate the gap size. The typical steps of an image analysis are reported in Figure S2. Thus, the average size of the NPs, and the mode of their gap size, was calculated (Figure S3).

### 2.5. Raman Analysis

4-mercaptobenzoic acid (4-MBA) was exploited as a probe to investigate the SERS performances of the synthetized substrates. Each sample was incubated in a 1 µM 4-MBA ethanolic solution (1 mL volume) for 30 min, and afterwards, was gently rinsed in ethanol (1 mL volume) three times. After removing the ethanol, the samples were left to dry in air. A Raman microscope (Qontor Renishaw—UK) was employed for the Raman measurements, using a 532-nm laser excitation wavelength. A streamline configuration was exploited to map the whole surface of the SERS substrate: the laser spot was modified to a line of several microns of length under which the sample was scanned. The correlation of the acquired signal to the specific sample coordinates was possible thanks to the synchronization of the stage motion and the change of the illuminated area of the CCD. The spectra acquisition was performed through a 10× objective and by using 4 s as the exposure time, with a laser power of 1 mW. SERS maps were acquired on a 2 × 2 mm^2^ area with a step size of 50 μm (corresponding to an average of 1400 spectra for each map).

## 3. Results and Discussion

### 3.1. Optical and Morphological Characterization

**Figure 1:**
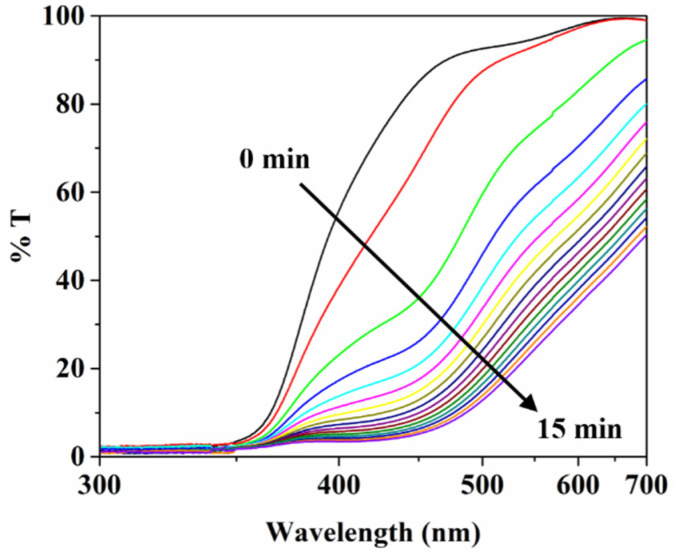
UV–Vis transmittance spectra of the chip showing the plasmonic dip evolution from 0 min to 15 min, using a 10 mM AgNO_3_ solution at 20 °C with a flow rate of 1 mL/min. Each spectrum was collected every minute.

The fabrication of the pSi substrates decorated with Ag-NPs was performed, exploiting the reactivity of the pSi layer to reduce the silver cations from the AgNO_3_ precursor solution [39,45]. Such a synthesis method has been widely used as a static approach thanks to its simplicity [46]; nevertheless, it does not provide optimal control over the reaction outcome, especially concerning the evolution of the synthesis reaction [14]. To improve the monitoring of the synthesis of the Ag-NPs, a microfluidic approach was exploited. The AgNO_3_ solution was flushed into a microfluidic chip, integrating a pSi membrane featuring an average pore diameter of 25 nm and a pore-size distribution between 10 and 40 nm (Figure S4), and the reaction was monitored in real time by UV–Vis transmittance spectroscopy. The black curve in Figure 1 shows the transmittance spectrum of the microfluidic chip filled with water that was used as a reference. As soon as the silver nitrate solution is injected in the microfluidic chamber, promoting the NP synthesis, the emergence of a spectral dip, located around 400–450 nm, is noticed (red curve), becoming more evident as the reaction proceeds over time. The appearance and the evolution of such a spectral feature is due to the rise of the LSPRs, thus proving the formation of the silver NPs. In detail, a shift towards a higher wavelength, as well as a broadening of the dip, are observed for longer times, due both to the increasing size and the reduced interparticle gap of the NPs. The reaction typically reaches a saturation regime in 15 min. After the quenching of the reaction by water injection, the morphological characterization of the synthesized Ag-decorated substrates was performed through FESEM. The acquired micrographs were then processed by the image analysis routine (detailed above) to analyse the main morphological features, such as the size and the inter-particle distance distributions of the synthetized NPs.

Table 1 details the tested combinations of the three main synthesis parameters (silver precursor concentration, temperature, and flow rate). As reference, the same conditions were tested using a static approach, by attaching a pSi–PDMS membrane to the inner wall of a plastic cuvette that was subsequently filled with a silver nitrate solution.

**Figure 2:**
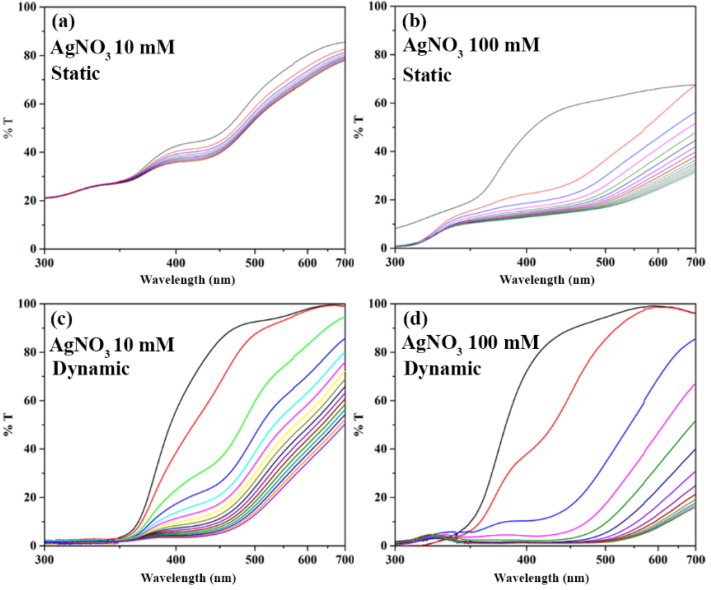
UV–Vis transmittance spectra of the chip showing the plasmonic dip evolution from 0 min. (black curve, water filled chip) to the final reaction time, with a 1 min step (the same colour sequence is used in all graphs, starting from the red curve, corresponding to the silver precursor injection) for different synthesis conditions: (**a**) static synthesis using a 10 mM AgNO_3_ solution at 20 °C; (**b**) static synthesis using a 100 mM AgNO_3_ solution at 20 °C; (**c**) dynamic synthesis using a 10 mM AgNO_3_ solution at 20 °C with a flow rate of 1 mL/min; (**d**) dynamic synthesis using a 100 mM AgNO_3_ solution at 20 °C with a flow rate of 1 mL/min.

The differences between the dynamic and static approaches are already evidenced by the optical characterization. Figure 2 shows the comparison between the UV–Vis transmittance spectra of the samples obtained, at 20 °C, using a 10 mM (Figure 2a,c) and 100 mM (Figure 2b,d) silver nitrate solution, by exploiting the static (Figure 2a,b) and the dynamic (Figure 2c,d) protocols. A first difference is evident by comparing the respective spectral evolutions. Concerning the static protocol, the reaction seems to start quickly, yielding a marked transmittance variation, but it then ends abruptly, reaching a saturation regime (Figure 2a,b). Such results can be explained by taking into account the mass transfer of AgNO_3_ from the bulk solution to the active surface of the pSi layer, where the formation of the Ag nuclei starts. The transfer rate is, indeed, regulated by the diffusion of the silver cations towards the pSi layer and, particularly in static conditions, this may affect the NPs’ synthesis, favouring their growth at the most reactive sites of the pSi surface, thus resulting in a more heterogeneous size distribution [26]. On the contrary, in dynamic conditions, the mass transfer of the AgNO_3_ is controlled by the flow rate, which can be tuned to obtain the desired NPs morphology, similarly as recorded for the in-flow synthesis of Au NPs [26]. Actually, a more homogeneous NPs growth can be achieved by the dynamic approach, probably due to the non-diffusion-limited growth of the Ag nuclei on the pSi surface, as suggested by the continuous evolution of the LSPRs over time (Figure 2c,d), in contrast to the early saturation observed in the static syntheses. Moreover, regardless of the adopted protocol (dynamic or static), the spectra in Figure 2 reveal that the silver nitrate concentration affects the evolution of the LSPRs. Indeed, the plasmonic dip appears to be broader and red-shifted in the spectra obtained by using a 100 mM AgNO_3_ solution, rather than a 10 mM one, that, as stated before, should indicate the production of larger and more densely distributed NPs. Finally, it is worth noticing that, by means of the 100 mM silver nitrate solution, a faster reaction rate is attained and, therefore, the saturation regime is reached earlier.

**Figure 3:**
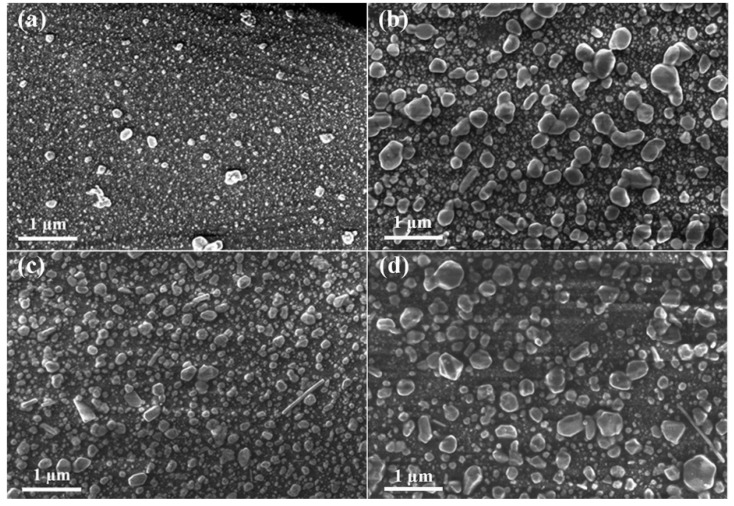
FESEM micrographs of samples fabricated using different synthesis conditions: (**a**) static synthesis using a 10 mM AgNO_3_ solution at 20 °C; (**b**) static synthesis using a 100 mM AgNO_3_ solution at 20 °C; (**c**) dynamic synthesis using a 10 mM AgNO_3_ solution at 20 °C with a flow rate of 1 mL/min; (**d**) dynamic synthesis using a 100 mM AgNO_3_ solution at 20 °C with a flow rate of 1 mL/min.

**Figure 4:**
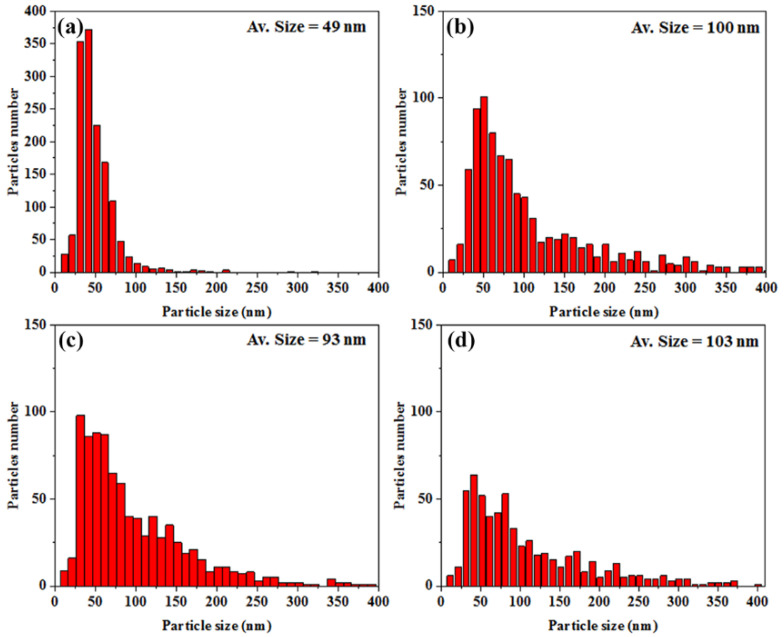
Particle size distribution of samples fabricated using different synthesis conditions: (**a**) static synthesis using a 10 mM AgNO_3_ solution at 20 °C; (**b**) static synthesis using a 100 mM AgNO_3_ solution at 20 °C; (**c**) dynamic synthesis using a 10 mM AgNO_3_ solution at 20 °C with a flow rate of 1 mL/min; (**d**) dynamic synthesis using a 100 mM AgNO_3_ solution at 20 °C with a flow rate of 1 mL/min. The data were obtained by means of the MATLAB routine.

Figure 3 displays the FESEM micrographs acquired on the same set of samples. Different NPs dimensions and size distributions are observed according to the different synthesis processes. By comparing the substrates obtained with the static (Figure 3a,b) and the dynamic approaches (Figure 3c,d), a more uniform distribution of the Ag NPs obtained in dynamic conditions is observable. Furthermore, in both cases, the use of a 100 mM silver nitrate concentration led to an increase in particle size. Such results are in accordance with the UV–Vis transmittance analysis. In particular, the detrimental effect of the static reaction protocol can be observed in Figure 3b, where the NPs’ distribution appears to be very heterogeneous. In more detail, the enhanced reaction rate, coupled with the slow diffusion of the AgNO_3_, led to an uneven growth of the Ag nuclei, which caused the production of larger, but isolated, silver nanoparticles. The results of the image analysis, reported in Figure 4, support such an interpretation. A broader NPs size distribution and an increased average size are indeed obtained for the samples synthesized with the 100 mM silver nitrate concentration. Interestingly, at such high silver nitrate concentrations, the differences between the particle size distributions obtained by the static and dynamic syntheses are reduced, suggesting that the quick consumption of silver cations at the pSi surface can still cause local inhomogeneities in silver nitrate concentrations and, thus, a higher polydispersity, even when the precursor is continuously refreshed.

For what concerns the computed inter-particle gap distributions, the mode was calculated instead of the average. Indeed, the image analysis did not show a normal distribution of the gap size (Figure S3), causing the average gap size to be shifted towards higher values due to the contributions of a minor fraction of gaps featuring large dimensions. This could cause a misrepresentation of the data, so the mode of the inter-particle size distribution was calculated to provide the most-represented gap size in the distribution. The analysis of these results reveals that smaller gaps are obtained by using the dynamic approach, at 10 mM AgNO_3_, or the static protocol, at 100 mM AgNO_3_, even if, in this latter case, the size distribution is quite broad. Therefore, the dynamic approach at the lowest tested concentration of silver nitrate is expected to provide more effective nanostructures, since the greatest SERS enhancements are obtained by hot-spots created by small inter-particle gaps [20]. Again, quite-large gaps are obtained by the dynamic approach if the concentration is raised to 100 mM AgNO_3_.

**Figure 5:**
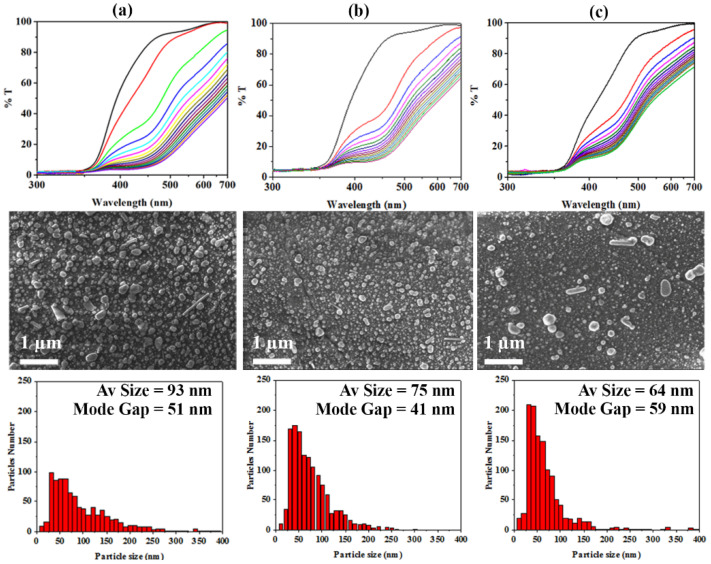
UV–Vis transmittance spectra showing the plasmonic dip evolution from 0 min. (black curve, water filled chip) to the final reaction time, with 1 min step (the same colour sequence is used in all graphs, starting from the red curve, corresponding to the silver precursor injection), FESEM micrographs, and particle size distribution of the samples synthetized in dynamic conditions using a 10 mM AgNO_3_ at 20 °C employing a flow rate of (**a**) 1 mL/min, (**b**) 2 mL/min, and (**c**) 3 mL/min.

After the direct comparison of the static and dynamic approaches, the effects of each synthesis parameter on the reaction outcome were carefully analysed. At first, the influence of the flow rate, ranging from 1 mL/min to 3 mL/min, was investigated, and the related transmittance spectra and FESEM micrographs, alongside the results obtained from the image analysis, are shown in Figure 5.

From the analysis of the transmittance spectra, it can be inferred that an increase in the flow rate leads to the production of smaller NPs with a low polydispersity degree: the plasmonic dip becomes much narrower and blue-shifted as the flow rate rises. Indeed, from the FESEM micrographs, a trend in the NP sizes is observable, depending on the effect of the flow rate increase, inducing the formation of smaller as well as more homogeneous particles, as already reported for in-flow growth of AuNPs [25,26]. Indeed, this behaviour is confirmed by the image analysis that reveals a smaller average size and a more uniform particle size distribution as the flow rate increases. The described trend is evident in Figure S5a, which plots the average NPs size obtained using a 10 mM AgNO_3_ concentration at 20 °C against the flow rate. The error bars, corresponding to the standard deviation of the average NPs size calculated for three samples synthesized under the same synthesis conditions, demonstrate a quite-good inter-sample reproducibility. The inter-particle gap size is influenced by the formation of smaller NPs. Indeed, increasing the flow rate from 1 mL/min to 2 mL/min reduces both the average size and the inter-particle gap. Instead, with the highest tested flow rate, the size is further reduced but the gap is slightly increased, probably because of the decreased coverage of the surface and/or the more-dispersed nucleation of the AgNPs on the pSi surface. 

**Figure 6:**
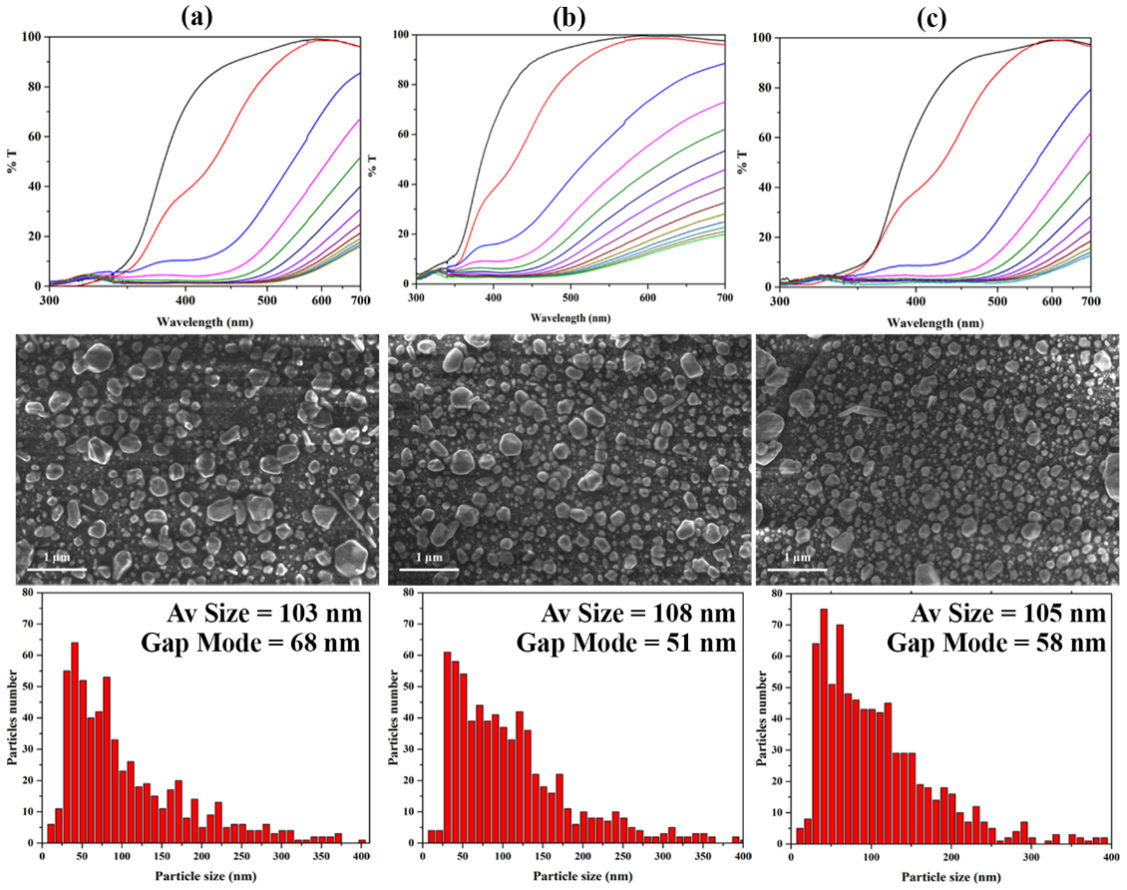
UV–Vis transmittance spectra showing the plasmonic dip evolution from 0 min. (black curve, water filled chip) to the final reaction time, with 1 min step (the same colour sequence is used in all graphs, starting from the red curve, corresponding to the silver precursor injection), FESEM micrographs, and particle size distributions of the samples synthetized in dynamic conditions using a 100 mM AgNO_3_ at 20 °C, employing a flow rate of (**a**) 1 mL/min, (**b**) 2 mL/min, and (**c**) 3 mL/min.

Once the effect of the flow rate was studied, a careful analysis was directed to the influence of the concentration of the silver nitrate in solution. Furthermore, the rise of the concentration was coupled with a variation of the flow rate in order to understand their combined effect on the synthetized NPs’ morphology. The results are shown in Figure 6. As stated before, the increase of the concentration up to 100 mM led to a faster reaction rate, allowing for the saturation regime to be reached in a shorter period of time, and for the production of NPs with larger diameters. Indeed, from the comparison of these transmittance spectra (Figure 6, 100 mM AgNO_3_ concentration at different flow rates) with the ones belonging to the samples obtained using a 10 mM silver nitrate solution (Figure 5), it is clearly noticeable that the plasmonic dip appears to be much broader and red-shifted, thus indicating that NPs with larger diameters and higher polydispersities should be expected. As for the previous case, the FESEM micrographs confirm the expectations, given that the produced samples feature larger AgNPs as well as a wider particle-size distribution. Nevertheless, the effect of an increment of the flow rate is not as evident as for the previous series of samples. Indeed, the NPs’ size distribution, as well as the average size and inter-particle gap modes, computed by the image analysis routine, do not appear to be significantly affected by the increase of the flow rate, as can also be inferred by the summary plot in Figure S5b, which shows the NPs’ size variation versus a flow rate increase. Nevertheless, a more homogeneous distribution and an overall decrease of the NPs’ size can be appreciated in the FESEM micrographs of the sample obtained using a 3-mL/min flow rate (Figure 6c), compared to the one obtained with a 1-mL/min flow rate (Figure 6a). These results show that the effect of an increment of the flow rate can be partially overcome by an increase of the silver nitrate concentration.

**Figure 7:**
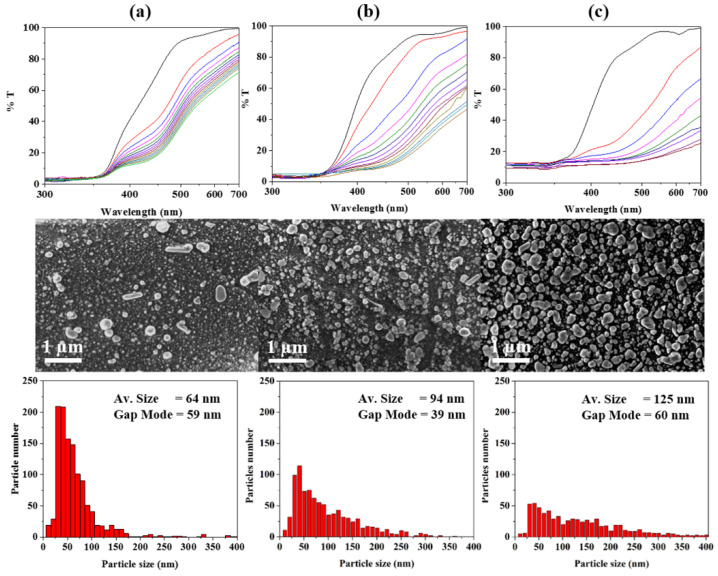
UV–Vis transmittance spectra showing the plasmonic dip evolution from 0 min. (black curve, water filled chip) to the final reaction time, with 1 min step (the same colour sequence is used in all graphs, starting from the red curve, corresponding to the silver precursor injection), FESEM micrographs, and particle size distributions of the samples synthetized using a 10 mM AgNO_3_ solution with a flow rate of 3 mL/min at a temperature of (**a**) 20 °C, (**b**) 40 °C, and (**c**) 60 °C.

At last, the effect of temperature, ranging from 20 °C to 60 °C, combined with both the concentration and flow rate, was investigated. The results show that the temperature is the main factor affecting both the kinetics of the reaction and the morphological characteristics of the obtained samples. Indeed, at 60 °C, a strong acceleration of the reaction rate is observed for both the samples produced using 10 mM and 100 mM silver nitrate concentrations (Figures S6 and S7). Furthermore, the produced NPs feature larger average diameters as well as higher polydispersities if compared to the samples obtained at lower temperatures. Such results are well emphasized by the samples obtained using the 10 mM AgNO_3_ solution, flushed at 3 mL/min, at different temperatures (Figure 7). It is indeed noticeable that the average size of the NPs, as well as the heterogeneity of the samples, seems to increase as the temperature is raised from 20 °C to 60 °C. Such a result, clearly highlighted in the plot of Figure S5c, is probably due to the strong increment of the reaction rate caused by the temperature increase, which inhibits a uniform growth of the particles. This behaviour is also reflected in the transmittance spectra recorded during the microfluidic synthesis. This effect is even enhanced if a 100 mM AgNO_3_ concentration is used (data not shown). Finally, it should be noted that, despite the reduced intra-sample homogeneity, an increase in the temperature or of the silver precursor concentration does not worsen the inter-sample repeatability (Figure S5). Indeed, the relative standard deviations (%RSD) calculated based on the replicates prepared under the same synthesis conditions, always range between 3 and 11%. Interestingly, such values are lower than the ones associated with the replicates obtained by a static approach (i.e., average NPs sizes of 53 nm ± 25% and 99 nm ± 15% when a 10 mM or 100 mM AgNO_3_ concentration is used at 20 °C), suggesting a moderate improvement of the synthesis reproducibility thanks to the exploitation of a microfluidic protocol.

### 3.2. SERS Analysis

The metal–dielectric nanostructures were used as solid SERS substrates, exploiting their plasmonic response. In detail, the SERS efficiency and reproducibility of the differently obtained substrates were explored by using 4-MBA, as described in the experimental section, as the Raman probe. The whole area of the samples was analysed exploiting the streamline configuration of the Raman spectrometer, applying a 1400-point grid map. Finally, the efficiency and the reproducibility of the SERS signal were evaluated, taking advantage of the band centred at 1077 cm^−1^_,_ arising from the ν_12_ vibration of the 4-MBA aromatic ring [47]. Indeed, its spectral sharpness and distance from the other vibrational components makes it ideal for the evaluation of the integrated area, which gives a representative indication of the sample’s efficiency and its point-to-point fluctuation within the substrate surface, measured through the relative standard deviation (RSD%).

As stated in the previous paragraph, the variation of the synthesis parameters, particularly concerning those that strongly enhance the reaction rate, can cause deep differences in the nucleation and growth of the silver seeds, leading to a large variety of particle and inter-particle distance distributions and, therefore, to a large range of intensity and RSD% values. Furthermore, to visually represent the intra-substrate SERS signal fluctuation, a false colour map of the integrated area of the 1077-cm^−1^ peak was obtained for each sample. The results of the assessment of the SERS performance of the synthesized substrates were, finally, inspected while taking into account the morphological parameters computed by the FESEM micrographs analysis indicated in the previous paragraph. 

**Figure 8:**
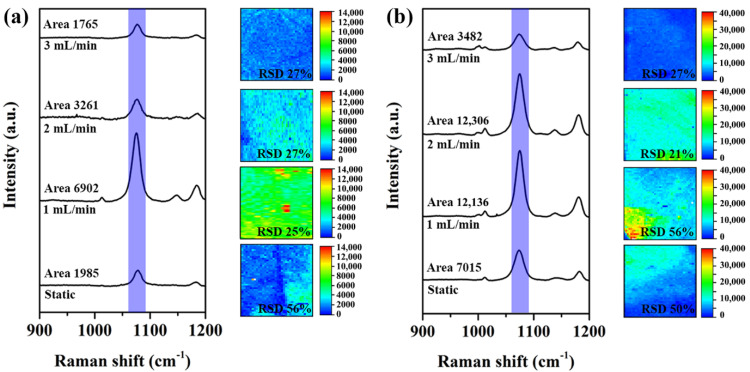
SERS efficiency evaluation and false colour intensity maps for the samples synthetized using (**a**) 10 mM AgNO_3_ at 20 °C with different flow rates, and (**b**) 100 mM AgNO_3_ at 20 °C with different flow rates. The spectra and maps corresponding to the samples prepared in static conditions are shown at the bottom of each SERS graph. The SERS spectra in this figure are the average of all the spectra collected by the mapping.

At first, the static and the dynamic conditions were tested and compared. The samples obtained by flushing 1 mL/min of precursor solution returned the highest SERS efficiency at a 10 mM concentration. Even if, in the previous section, the FESEM micrographs showed a slightly increased homogeneity of the samples synthetized exploiting the dynamic approach (Figure 3), the RSD% was halved with the in-flow synthesis (Figure 8a). On the other side, a further increase of the flow rate of up to 3 mL/min led to the production of smaller NPs, which negatively affected the SERS efficiency of the substrates, whereas the RSD% was not further improved. It should be noted that, even if few large NPs are present at the highest tested flow (Figure 5c), from the application point of view, such isolated particles do not significantly contribute to the detected SERS response. Actually, large isolated particles cannot yield Raman hot-spots related to inter-particle surface plasmons, which provide most of the SERS enhancement.

Afterwards, the set of substrates, fabricated by increasing the silver nitrate concentration up to 100 mM and with different flow-rate values (Figure 8b), was analysed. As stated before, the AgNO_3_ concentration increase led to a faster reaction rate and to the production of larger NPs; therefore, a variation in the SERS performances of the substrates was expected. Indeed, the average SERS intensity displayed by this set of substrates was greater than the one related to the samples obtained using a 10 mM AgNO_3_ solution (Figure 8a). Once more, the sample prepared in static conditions provided a high RSD% value, due to the wide size-distribution of the resulting NPs. On the contrary, the dynamic approach yielded a decrease of the RSD% only starting from 2 mL/min, probably because a higher flow rate is required to mitigate the influence of a higher concentration of the silver precursor on the growth of the nanoparticles. Such results are in good agreement with the information collected from the related FESEM micrographs (Figure 6), which show that smaller but more uniformly distributed Ag-NPs are produced as the flow rate increases. Consistently with such findings, and similarly to the previous case, the SERS efficiency diminishes if the flow rate is too high, as is possible to notice from the SERS analysis of the samples obtained by flowing the precursor at 3 mL/min (Figure 8b).

**Figure 9:**
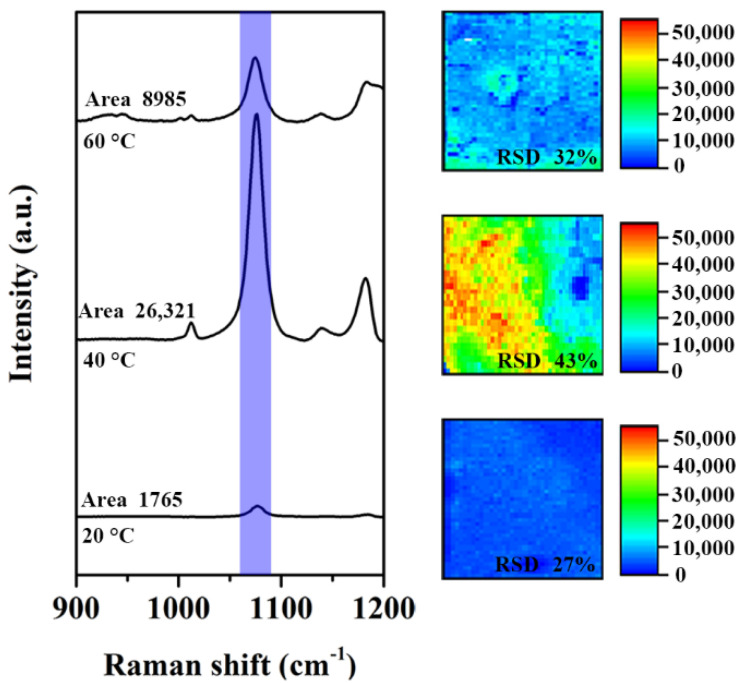
SERS efficiency evaluation and false colour intensity maps for the samples synthetized using 10 mM AgNO_3_ combined with a 3-mL/min flow rate, at different temperatures. The SERS spectra in this figure are the average of all the spectra collected by the mapping.

At last, the effect of temperature was investigated by analysing the samples obtained by flowing a 10 mM AgNO_3_ solution at 3 mL/min and by changing the temperature from 20 °C to 60 °C. As for the previous experiment, the enhanced reaction rate, in this case due to the temperature rise, led to the production of more heterogeneous samples, so the RSD% increased (Figure 9). On the other side, concerning the SERS efficiency, the sample obtained at 60 °C showed an enhanced intensity of the reference Raman peak, but the best performances were provided by the substrate produced at 40 °C, whose average spectrum displayed the highest peak intensity of the whole dataset, with a value of about 15 times the average area of the sample obtained at 20 °C.

To summarize, a correlation between the variation of the synthesis parameters and the SERS response was found for the samples synthesized at 10 mM concentration and low temperatures, as detailed in the previous paragraph. Instead, an increase of temperature combined with an increase of the AgNO_3_ concentration proves to be detrimental, as the uncontrolled growth of the Ag-NPs due to the strong increment of the reaction rate negatively affects the homogeneity of the synthesis. Indeed, the obtained samples displayed complex morphologies, showing several populations of NPs of different sizes. In such a framework, the correlation between the morphology and the SERS performances of the obtained substrates can be quite challenging. A good SERS substrate must feature a high Raman signal and a low/moderate RSD%, so that samples synthesized with the static approach show poor performances. On the other side, as usually reported in the literature [48], intense Raman signals are yielded by nanostructures where a low inter-particle gap can be inferred. This is, for instance, the case of the most efficient substrate, obtained using 10 mM silver nitrate, 40 °C, and a strong flow rate (3 mL/min) to compensate for the driving effect of temperature. Unfortunately, this sample is featured by a quite-high polydispersity of the AgNPs on the surface that is reflected on the relatively high RSD%. Then, a good compromise can be reached by the nanostructures obtained at 20 °C, by flushing a 100 mM AgNO_3_ solution at 2 mL/min.

## 4. Conclusions

In this work, Ag-NPs were synthesized in flow on porous silicon–PDMS membranes and were carefully characterized. The correlation between the synthesis parameters variation and the morphologies of the produced silver nanoparticles assemblies was investigated by using a microfluidic approach. To this aim, the pSi–PDMS membrane was integrated in a microfluidic cell, in which a silver nitrate solution was injected. Moreover, the exploited configuration allowed for the real-time monitoring of the reaction by means of UV–Vis spectroscopy. Three different synthesis parameters were selected and varied in order to determine their effect on the optical and morphological characteristics of the obtained samples: (i) the silver precursor concentration, (ii) the temperature, and (iii) the flow rate.

It was observed that the temperature increase led to a strong acceleration of the reaction rate and to the production of larger NPs, but, on the other side, the polydispersity of the obtained samples was quite high, probably due to the fast kinetics of the reaction. A similar but weaker effect was observed by increasing the silver precursor concentration up to 100 mM, which, in most of the cases, caused an increment of the nanoparticles’ dimensions. Lastly, the flow rate was proven to be the least-effective parameter, as its impact was often overcome by the temperature and concentration variation. Nevertheless, it displayed an opposite effect with respect to the other two parameters, as the nanoparticles grew smaller and more uniform when increasing the flow rate. 

The nanostructures were finally tested as solid SERS substrates using 4-MBA as a probe molecule, analysing the correlation between the synthesis parameters, the morphologies, and the SERS performances of the related substrates. In particular, the results obtained by analysing the samples fabricated at low silver nitrate concentrations, as well as the samples prepared at 20 °C with a 100 mM silver nitrate solution, showed a good correlation between the SERS data and the morphological characteristics. On the other side, the combined rise of the silver precursor concentration and the temperature led to an uncontrolled increase of the reaction rate, causing the production of very heterogeneous samples with complex morphologies. The developed microfluidic protocol has, thus, proven to be effective for the real-time monitoring of the synthesis reaction, allowing us to isolate the effects of each synthesis parameter and to identify how they should be combined to achieve the fabrication of reliable SERS substrates.

## Figures and Tables

**Table 1:** Synthesis parameters along with the corresponding tested values.

Synthesis Parameter	Tested Values
[AgNO_3_]	10–100 mM
Temperature	20–40–60 °C
Flow Rate	0–1–2–3 mL/min

## Data Availability

The data presented in this study are available on request from the corresponding author.

## Supplementary Materials

The following are available online at https://www.mdpi.com/article/10.3390/bios11120520/s1, Figure S1. (a) Overview of the complete experimental setup for the dynamic synthesis; (b) and (c) microfluidic chamber hosting the ultrathin pSi layer. A syringe pump drives the injection of the silver precursor solution into the microfluidic chamber located inside the UV-Vis spectrophotometer. The temperature of the precursor solution is controlled by means of a thermostatic bath. Figure S2. An example of image analysis—(a) FESEM micrographs of a selected sample; (b) identification of each na-noparticle; (c) traces corresponding to the semi-distances between nanoparticles. Figure S3. Size distribution of the inter-particle gaps. Figure S4. Top-view FESEM micrograph of the porous silicon membrane after the detachment from the original silicon substrate. Figure S5. Effect on the average AgNP size of (a) the flow rate at 10 mM AgNO3 concentration and 20 °C (b) the flow rate at 100 mM AgNO3 concentration and 20 °C (c) the temperature at 10 mM AgNO3 concentration and 3 mL/min flow rate. The error bars represent the standard deviation obtained considering three replicas for each synthesis condi-tion. Figure S6. UV-Vis transmittance spectra showing the plasmonic dip evolution from 0 min. (black curve, water filled chip) to the final reaction time, with a 1 min step (the same colour sequence is used in all graphs, starting from the red curve, corresponding to the silver precursor injection), particle size distribution, and FESEM micrographs of the samples synthetized in dynamic conditions using a 10 mM AgNO3 at 60 °C employing a flow rate of (a) 1 mL/min (b) 2 mL/min (c) 3 mL/min. Figure S7. UV-Vis transmittance spectra showing the plasmonic dip evolution from 0 min. (black curve, water filled chip) to the final reaction time, with a 1 min step (the same colour sequence is used in all graphs, starting from the red curve, corresponding to the silver precursor injection), particle size distribution, and FESEM micrographs of the samples synthetized in dynamic conditions using a 100 mM AgNO3 at 60 °C employing a flow rate of (a) 1 mL/min (b) 2 mL/min (c) 3 mL/min.
