# Supplementary material for: Real-Time Monitoring of the In Situ Microfluidic Synthesis of Ag Nanoparticles on Solid Substrate for Reliable SERS Detection
Source: Biosensors (Basel). 2021 Dec 16;11(12):520. doi: 10.3390/bios11120520 (PMC8699179; doi:10.3390/bios11120520)
Supplement: Supplementary file 1 [file biosensors-11-00520-s001.zip › biosensors-1438793-sup.pdf]

biosensors

MDPI

Article

Real-Time Monitoring of the In Situ Microfluidic Synthesis of Ag Nanoparticles on Solid Substrate for Reliable SERS Detection

Niccolò Paccotti [1,†], Alessandro Chiadò [1,2,†], Chiara Novara [1,*], Paola Rivolo [1], Daniel Montesi [1], Francesco Geobaldo [1] and Fabrizio Giorgis [1]

[1] Department of Applied Science and Technology, Politecnico di Torino, C.so Duca degli Abruzzi 24, 10129 Turin, Italy; niccolo.paccotti@polito.it (N.P.); alessandro.chiado@polito.it (A.C.); paola.rivolo@polito.it (P.R.); daniel.montesi@polito.it (D.M.); francesco.geobaldo@polito.it (F.G.); fabrizio.giorgis@polito.it (F.G.)

[2] Center for Sustainable Future Technologies @Polito, Istituto Italiano di Tecnologia, Corso Trento 21, Torino 10129, Italy

* Correspondence: chiara.novara@polito.it

† These authors contributed equally to this work.

Citation: Paccotti, N.; Chiadò, A.; Novara, C.; Rivolo, P.; Montesi, D.; Geobaldo, F.; Giorgis, F. Real-Time Monitoring of the In Situ Microfluidic Synthesis of Ag Nanoparticles on Solid Substrate for Reliable SERS Detection. *Biosensors* **2021**, *11*, 520. https://doi.org/10.3390/bios11120520

Received: date
Accepted: date
Published: date

Publisher's Note: MDPI stays neutral with regard to jurisdictional claims in published maps and institutional affiliations.

Copyright: © 2021 by the authors. Submitted for possible open access publication under the terms and conditions of the Creative Commons Attribution (CC BY) license (https://creativecommons.org/licenses/by/4.0/).

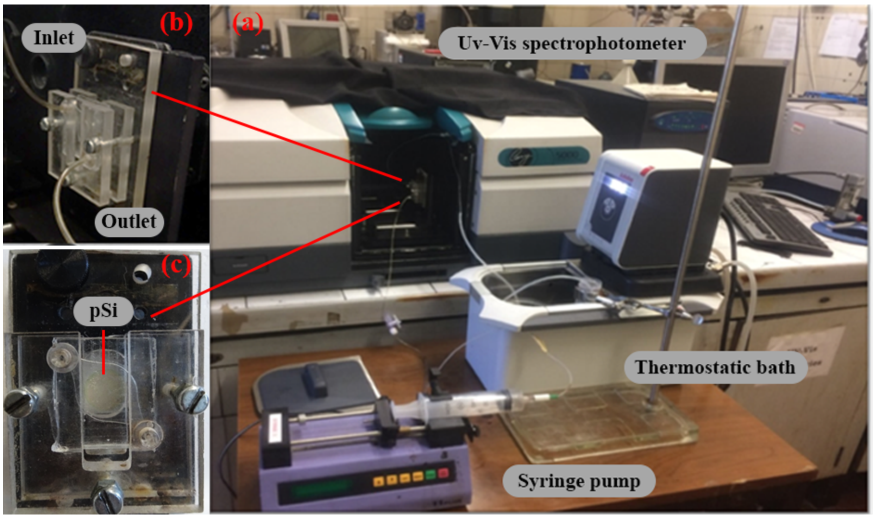

Figure S1. a) Overview of the complete experimental setup for the dynamic synthesis; b) and c) microfluidic chamber hosting the ultrathin pSi layer. A syringe pump drives the injection of the silver precursor solution into the microfluidic chamber located inside the UV-Vis spectrophotometer. The temperature of the precursor solution is controlled by means of a thermostatic bath.

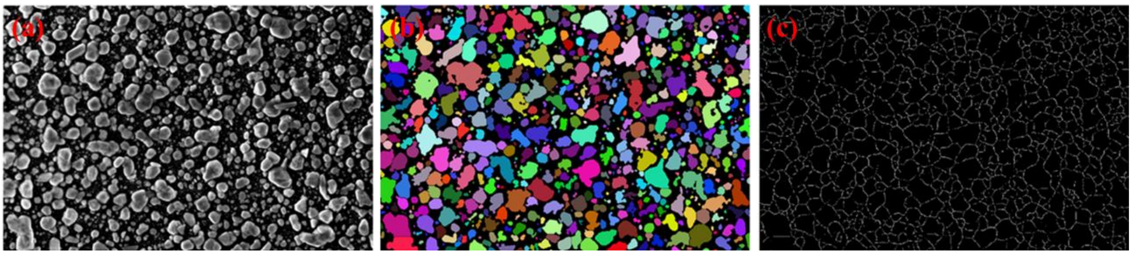

Figure S2. An example of image analysis – a) FESEM micrographs of a selected sample; b) identification of each nanoparticle; c) traces corresponding to the semi-distances between nanoparticles.

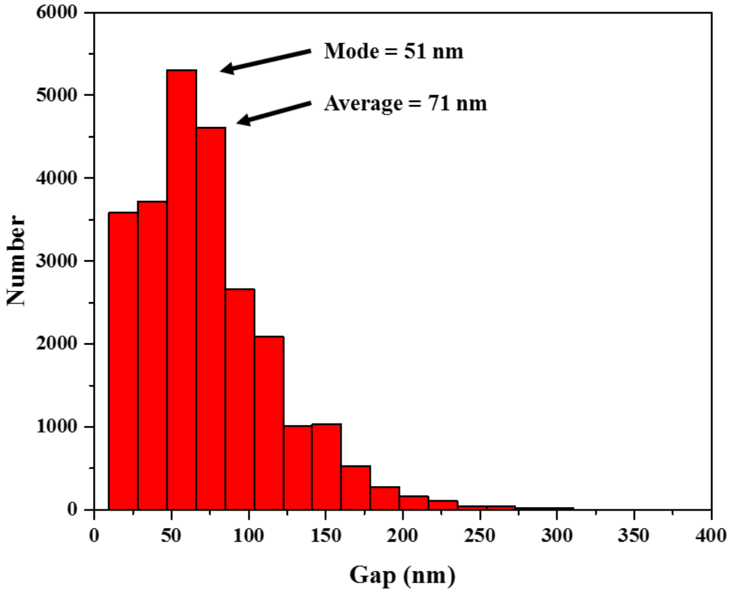

Figure S3. Size distribution of the inter-particle gaps.

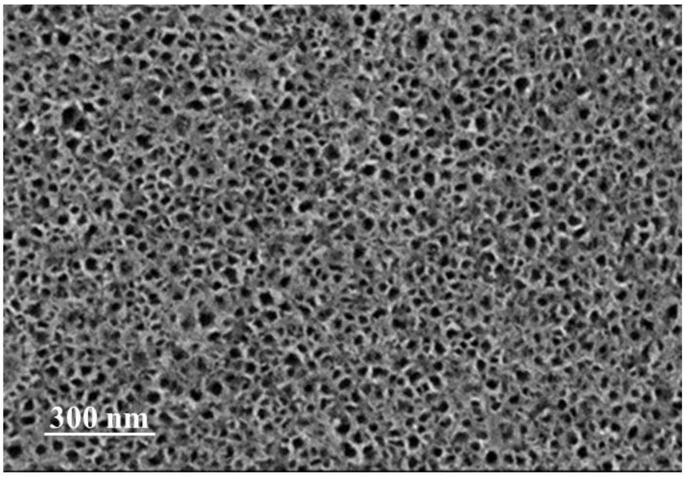

Figure S4. Top-view FESEM micrograph of the porous silicon membrane after the detachment from the original silicon substrate.

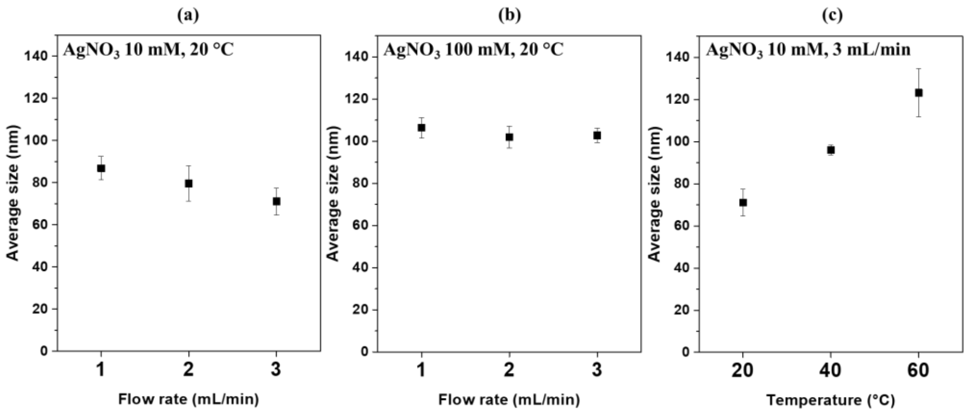

Figure S5. Effect on the average AgNP size of a) the flow rate at 10 mM $AgNO_3$ concentration and 20 °C b) the flow rate at 100 mM $AgNO_3$ concentration and 20 °C c) the temperature at 10 mM $AgNO_3$ concentration and 3 mL/min flow rate. The error bars represent the standard deviation obtained considering three replicas for each synthesis condition.

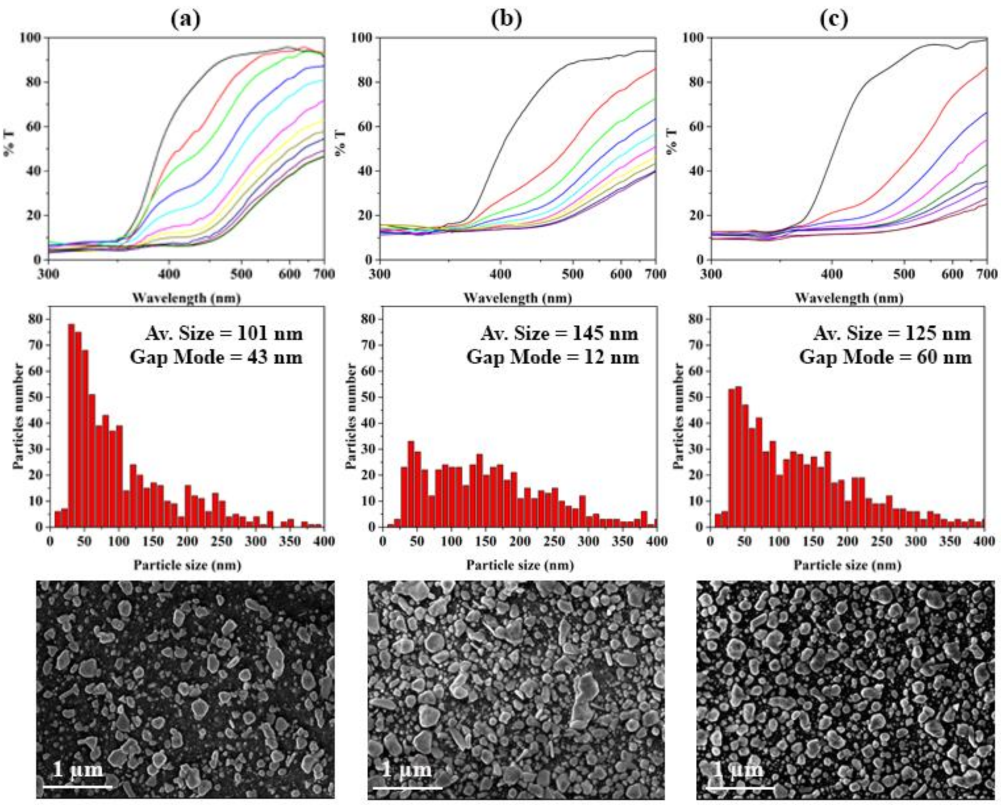

Figure S6. – UV-Vis transmittance spectra, particle size distribution, and FESEM micrographs of the samples synthetized in dynamic conditions using a 10 mM $AgNO_3$ at 60 °C employing a flow rate of a) 1 mL/min b) 2 mL/min c) 3 mL/min.

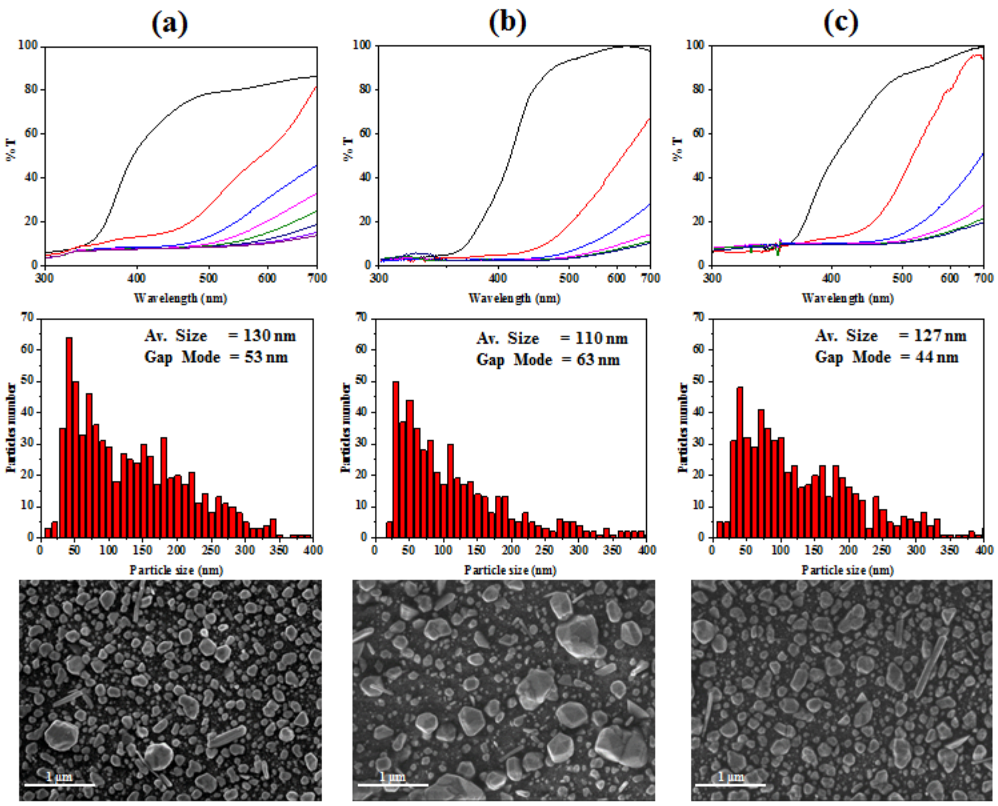

Figure S7. - UV-Vis transmittance spectra, particle size distribution, and FESEM micrographs of the samples synthetized in dynamic conditions using a 100 mM $AgNO_3$ at 60 °C employing a flow rate of a) 1 mL/min b) 2 mL/min c) 3 mL/min.
